# Use of Non-Destructive Ultrasonic Techniques as Characterization Tools for Different Varieties of Wine

**DOI:** 10.3390/s24134294

**Published:** 2024-07-02

**Authors:** José Ángel Corbacho, David Morcuende, Montaña Rufo, Jesús M. Paniagua, María Ángeles Ontalba, Antonio Jiménez

**Affiliations:** 1 Department of Applied Physics, IPROCAR Research Institute, Centro Universitario de Mérida, Universidad de Extremadura, Avda. Santa Teresa de Jornet, 38, 06800 Mérida, Spain; corbamer@unex.es; 2 Department of Food Technology, IPROCAR Research Institute, Universidad de Extremadura, Avenida de la Universidad s/n, 10003 Cáceres, Spain; demorcuen@unex.es; 3 Department of Applied Physics, IPROCAR Research Institute, School of Technology, Universidad de Extremadura, Avenida de la Universidad s/n, 10003 Cáceres, Spain; paniagua@unex.es (J.M.P.); ontalba@unex.es (M.Á.O.); ajimenez@unex.es (A.J.)

**Keywords:** ultrasound parameters, wine, ultrasonic transducers, ultrasonic pulse velocity, FFT, ultrasonic attenuation

## Abstract

In this work, we have verified how non-destructive ultrasonic evaluation allows for acoustically characterizing different varieties of wine. For this, a 3.5 MHz transducer has been used by means of an immersion technique in pulse-echo mode. The tests were performed at various temperatures in the range 14–18 °C. The evaluation has been carried out studying, on the one hand, conventional analysis parameters (velocity and attenuation) and, on the other, less conventional parameters (frequency components). The experimental study comprised two stages. In the first, the feasibility of the study was checked by inspecting twelve samples belonging to six varieties of red and white wine. The results showed clearly higher ultrasonic propagation velocity values in the red wine samples. In the second, nine samples of different monovarietal wine varieties (Grenache, Tempranillo and Cabernet Sauvignon) were analyzed. The results show how ultrasonic velocity makes it possible to unequivocally classify the grape variety used in winemaking with the Cabernet Sauvignon variety having the highest values and the Grenache the lowest. In addition, the wines of the Tempranillo variety are those that present higher values of the attenuation coefficient, and those from the Grenache variety transmit higher frequency waves.

## 1. Introduction

Wine, a complex and nuanced beverage, is the result of the fermentation of grapes, and it is characterized by its rich aroma, flavor profile, and alcoholic content. Chemically, wine comprises a diverse array of organic compounds, prominently including ethanol, water, sugars (such as glucose and fructose), organic acids (such as tartaric and malic acids), phenolic compounds (such as tannins and flavonoids), and volatile aroma compounds (such as esters and terpenes). The composition of wine can vary significantly depending on factors such as grape variety, growing conditions, fermentation process, and aging techniques. Understanding the composition of wine is essential for assessing its quality, flavor characteristics, and suitability for consumption [1].

Characterizing wine is crucial due to its wide range of implications. Firstly, the quality of wine is closely linked to its chemical and sensory composition, making characterization essential for producers, consumers, and critics alike [2,3]. Additionally, the economic implications of the wine industry are significant, as the demand for high-quality wines continues to rise. In terms of health, wine characterization can provide crucial information about its antioxidant content and other beneficial compounds [4]. However, the presence of fraud in the wine industry underscores the need for accurate characterization methods to ensure authenticity and transparency in the market. In this context, the research and development of advanced analytical techniques play a pivotal role in guaranteeing the quality, authenticity, and safety of wine products [5].

Traditional techniques used in wine characterization include sensory analysis, chromatography, spectrophotometry, and spectroscopy [6]. On one hand, these processes entail product destruction, while on the other, they yield chemical residues necessitating subsequent treatment, thereby posing waste management challenges. Moreover, conducting a comprehensive analysis requires significant time and incurs economic costs, which are compounded by the expensive nature of samples in this product category [7].

Non-invasive acoustic methods utilizing ultrasonic transducers offer a viable alternative to address the aforementioned challenges associated with conventional wine characterization techniques. Ultrasonic signals, renowned for their informational richness, excel in delineating the attributes of various substances, including liquids, semi-liquids, multiphase systems, opaque materials, and dense suspensions. Their ability to traverse container walls and chambers with minimal attenuation renders them indispensable for non-invasive, hygienic, precise, and cost-effective measurement systems, which are conducive to automated processes [8,9]. Moreover, the sensitivity of ultrasonic wave propagation to alterations in density, compressibility, turbidity, and viscosity facilitates the characterization of each wine’s unique properties [9].

This research builds upon the aforementioned line of inquiry. Its primary aim is to conduct an ultrasonic characterization of diverse wine types. Initially, the focus lies on distinguishing between white and red wines, which is followed by the analysis of single-varietal wines including Tempranillo, Grenache, and Cabernet Sauvignon—three widely utilized grape varieties in winemaking. In addition to conventional parameters like ultrasonic wave velocity and attenuation [10,11,12,13,14,15], this study introduces novel parameters associated with the frequency components present in sample transmission. These frequency parameters have already been used by the authors in other food substrates such as honey [16], edible vegetable oils [17], etc. Furthermore, the investigation extends to evaluating these parameters at two common consumption temperatures, 15 and 18 °C [18], acknowledging their documented influence on acoustic properties across various materials.

## 2. Materials and Methods

### 2.1. Samples

For the first part of the study, six commercial wine brands were acquired (two 750 mL bottles of each brand), all from the “Pago Los Balancines” winery, located in Badajoz, Spain. Three brands corresponded to red wines (Balancines Punto Rojo, Balancines Huno Blend, and Balancines Haragán) made from different grape varieties (tempranillo, syrah, tinta roriz, alicante bouschet, graciano, etc.) and with different aging times (between 5 and 15 months) in barrels. The other three corresponded to white wines (Balancines Punto Blanco, Balancines Huno White, and Alunado), also made from different grape varieties (sauvignon blanc, chardonnay, and viura). The vintages and alcohol content of the acquired wines were also different, specifically, between 2014 and 2019, and between 13.5% and 15%, respectively.

For the second part of the study, three 750 mL bottles of three commercial brands of red wine were acquired (Finca Antigua Tempranillo, Finca Antigua Cabernet Sauvignon, and Finca Antigua Garnacha), totaling nine bottles, from the Familia Martínez Bujanda winery (Cuenca, Spain). All bottles belonged to the same batch and aging year. As indicated by the brand of each wine, each of them is single varietal, meaning their production is carried out using the grape varieties Tempranillo, Cabernet Sauvignon, or Grenache, without any blending between them, as was the case with the samples acquired for the first part of the study. All samples had identical alcohol content (13.5%) and identical aging periods in American oak barrels (10 months).

All wine bottles acquired were stored in a thermostatic refrigerator at a constant temperature of (12.0 ± 0.2) °C until their opening. Table 1 lists the samples from the described bottles.

### 2.2. Ultrasonic Tests

The ultrasonic inspection followed a methodology similar to previous studies [17,19]. A volume of 105 mL was poured from each sample and transferred into a 100 mL Nessler tube. This tube was then carefully placed inside a 1000 mL tall form beaker filled with water at (12.0 ± 0.2) °C, ensuring that the free surfaces of both the water and wine in the Nessler tube were aligned, and avoiding any mixing between the liquids. Subsequently, the setup was gradually heated in a temperature-controlled room at (25.0 ± 0.5) °C. The water temperature was monitored using a Testo 925 digital thermometer with a resolution of 0.1 °C. Throughout the experiment, it was assumed that the temperatures of the wine and water were identical.

In the first part of the study, ultrasonic tests were conducted firstly at approximately 15 °C and then at 18 °C. The samples were inspected randomly, meaning they were not inspected in any specific order, alternating between white and red wines and always avoiding the consecutive inspection of two samples from the same wine brand. In the second part, the ultrasound inspections were carried out precisely at 15 and 18 °C. In both parts of the study, the time elapsed between the inspection at 15 °C and at 18 °C was approximately 15 min. This resulted in a total of 60 ultrasonic tests being conducted using the pulse-echo (PE) immersion technique to analyze the samples. The Olympus Model V381 piezoelectric transducer with a nominal frequency of 3.5 MHz was chosen for this purpose (key characteristics outlined in Table 2). During the inspection, the transducer was positioned at the top of the Nessler tube and submerged to a depth of about 1.5 cm in the wine. To ensure precise alignment with the tube’s bottom and isolate it from potential signals originating from the glass or metal structure, the transducer was mounted on a custom metal frame using elastic rubber fastenings. In this way, the possibility of a progressive increase in dissolved oxygen in the wine from the moment the bottle is opened is more limited in our case, as the Nessler tube is covered by the transducer and the custom-made metal device during the inspection and heating process. Some authors have estimated this increase under conditions similar to ours (although with direct exposure of the wine to the air), calculating it at 0.066 mg of oxygen × L^−1^ × min^−1^ (0.99 mg/L in the first 15 min) [20]. In the authors’ opinion, it probably does not represent relevant changes in the redox properties of the wine. The distance between the transducer’s surface and the tube’s bottom, acting as a reflecting mirror for the ultrasonic waves, was measured using a precision caliper with an accuracy of ±0.01 mm. This distance was determined to be 158.00 mm, ensuring that measurements were conducted in the far field (Fraunhofer region), where ultrasonic waves travel a minimum distance from emission to reception, which was 158.00 × 2 = 316.00 mm. This distance exceeded the length of the near field (Fresnel region) specified in Table 2, which was a deliberate choice critical for preventing wave interference phenomena from affecting the evaluation of parameters related to signal attenuation and frequency. Working in the far field mitigated potential interference issues, ensuring accurate parameter assessment [21].

**Figure 1:**
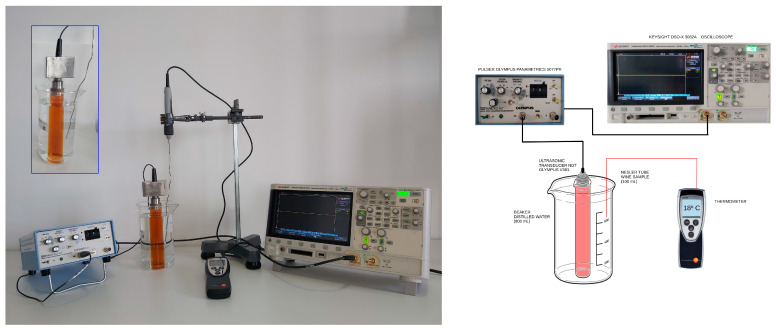
Picture and diagram of the experimental set-up for the ultrasonic tests.

Ultrasonic measurements were conducted using the Olympus-NDT Model 5077PR Pulser-Receiver, which was tasked with emitting and receiving signals from the piezoelectric transducer. This apparatus was linked to a KEYSIGHT InfiniiVision DSO-X 3032A oscilloscope, which was employed for recording and storing the A-scans with 16,000 data points in CSV format. Figure 1 illustrates the experimental setup utilized for these measurements. Throughout the ultrasonic examinations, an A-scan was captured from each of the 60 conducted tests. Each A-scan was acquired with a 1000 µs time interval (TI) on the oscilloscope. Thus, this interval encompassed the trigger pulse and the initial four complete echoes. As an illustration, Figure S1 displays the characteristic A-scan at 1000 µs corresponding to one of the examined samples. This A-scan reveals less intense signals, originating from successive reflections on the front and back surfaces of the bottom of the Nessler tube as well as on the lateral faces of the tube itself. Nevertheless, one can infer that the peak-to-peak amplitude of echo 1 is comparable to that of the wavefront reaching the transducer after experiencing only a reflection at the front surface of the Nessler tube bottom. This inference applies equally to each of the four echoes examined in the study. Moreover, as the wave propagation time through the wine is considerably longer than through the glass due to reflection–transmission processes at the tube bottom, none of the echoes depicted in Figure S1 would be significantly influenced by the preceding echoes.

As mentioned, the ultrasonic parameters under scrutiny included the following: propagation velocity (*UPV*); ultrasonic attenuation (*α*); and frequency values corresponding to 25%, 50%, 75%, and 99% (*FFT25*, *FFT50*, *FFT75*, and *FFT99*, respectively) of the frequency spectra obtained in the FFT (Fast Fourier Transform), which gives a frequency domain representation of the amplitude and phase of a continuous signal acquired in the domain time. These frequency percentiles are obtained from the corresponding cumulative frequency periodogram constructed from the FFT. These latter frequency values were obtained for both the entire signal and for each of the four echoes present in the A-scan.

The methods used to determine the ultrasonic parameters mentioned earlier closely follow those described in previous research [17,22]. Nonetheless, in the interest of clarity and conciseness, this study highlights only the variations from the procedures detailed in those works. The fundamental principles and techniques for calculation remain intact, ensuring consistency and comparability with the preceding research.

Regarding the calculation of *UPV* (propagation velocity), two methods were utilized. (1) Least squares fit: This approach involved conducting a least-squares fit to a straight line equation, correlating the known distance traveled by the ultrasonic signal with the corresponding time of flight for each of the four echoes. The time of arrival at maximum amplitude was utilized for this purpose. The slope of this fit yielded the propagation velocity, which was referred to as *UPV_lr_* [19]. (2) Cepstrum method: This calculation relied on the Fast Fourier Transform (FFT) of the A-scan. For example, Figure S2 demonstrates the FFT generated from the A-scan shown in Figure S1. The presence of periodic excitation (in this case, multiple echoes) appears as peaks in the spectrum at multiples of the fundamental frequency, resulting in superimposed spectra of the probe’s fundamental spectrum with evenly spaced peaks. The time of flight between echoes produced by successive reflections between the bottom of the Nessler tube and the transducer’s surface is determined from the distances of these peaks. Using the cepstrum method, the spectrum can be smoothed, and the period’s length can be directly determined. The cepstrum is generated by applying a Fast Fourier Transform (FFT) to the logarithmized spectrum [23,24]. Algorithmically, the cepstrum *C* is defined by Equation (1):

(1) $$C=FFT^{-1}\left\{\ln\left[FFT\left(A-scan\right)\right]\right\}$$

This second FFT directly reveals the time (*t*) elapsed between these excitations (time of flight between two consecutive echoes). By combining this information with the known distance (0.316 m) traveled by the ultrasonic wave between these echoes, the velocity *UPV_cepstrum_* could be directly calculated as *UPV_cepstrum_* = 0.316/*t*. As an illustrative example, Figure S3 demonstrates the cepstrum generated from the FFT of Figure S2. The associated errors for the determinations of *UPV_lr_* and *UPV_cepstrum_* were found to be approximately 0.032% and 0.012%, respectively. These relatively small errors highlight the accuracy and reliability of the velocity measurements obtained through these methods.

The attenuation *α* (in neper/m) was computed using Equation (2):

(2) $$\alpha =\frac{1}{2d}\ln\frac{A_{i}}{A_{j}}$$

where *A_i_* and *A_j_* represent the peak-to-peak amplitudes of echoes *i* and *j*, respectively, and 2*d* corresponds to the distance covered by the ultrasound wave between these two echoes. The determination of *α* was derived from the analysis of the four echoes obtained from the A-scan. The calculation of *α* entailed plotting ln(*A_i_*/*A_j_*) against 2*d* with the slope of this plot yielding the value of the attenuation [25]. It should be noted that the value obtained for *α* is closely associated with the inspection geometry, and its validity can only be compared with those obtained in samples sharing identical measurement geometry, as is the case with all the samples inspected in this study [17].

The determination of *FFT25*, *FFT50*, *FFT75*, and *FFT99* is predicated on the understanding that the ultrasonic frequencies transmitted through the samples may not precisely align with the nominal frequencies emitted by the transducer [22]. Hence, it is more meaningful to consider the frequencies that are actually transmitted and appear in the associated FFT for each A-scan. These frequencies in the FFT offer a more precise representation of the signal characteristics in the frequency domain. However, it is crucial to acknowledge a caveat stemming from the Nyquist theorem, which establishes the minimum sampling frequency *f’* required to accurately study any frequency signal *f* in the frequency domain, as depicted in Equation (3) [26]:

(3) f’≥2f

In our case (TI = 1000 μs), the value of *f’* is 16 MHz. Considering that the central frequency *f* of the utilized transducer is 3.5 MHz, the aforementioned value of *f’* unequivocally verifies Equation (3). As an illustration, Figure S4 displays the Fast Fourier Transforms (FFTs) corresponding to each of the four echoes present in the A-scan depicted in Figure S1. Three observations are pertinent to this matter: (1) Unlike what was observed in the FFT corresponding to the complete A-scan (see Figure S2), in the FFT corresponding to each of the four echoes, the characteristic periodic excitation is not apparent, as no other echo appears within the considered temporal interval of the A-scan. (2) Despite the transducer having a central frequency of 3.5⋅10^6^ Hz and a 6 dB bandwidth of 66.23%, the obtained FFTs do not exhibit a maximum amplitude at that frequency but rather at other frequencies that are significantly lower. (3) The normalized amplitude of the FFT corresponding to each echo decreases as the number of echoes considered increases as a consequence of the greater attenuation experienced by the ultrasonic waves traveling a greater distance. Using these FFTs, we constructed cumulative frequency periodograms, displaying the 25th, 50th, 75th, and 99th percentiles of the frequencies present in the received signals in each echo. In concrete terms, the *FFT50* of a particular echo would correspond to the minimum frequency value for which 50% of the total energy would have been received. That is to say, if the 50th percentile of the cumulative frequency was located at x Hz for a particular echo of a particular inspection, this would indicate that 50% of the received signals would have frequencies lower than x Hz. The same applies to *FFT25*, *FFT75* and *FFT99*. For illustrative purposes, Figure S5 displays the cumulative frequency periodograms generated from the FFTs presented in Figure S4. One final observation: In general, the values of *FFT25*, *FFT50*, *FFT75* and *FFT99*, corresponding to each echo, decrease as the number of echoes considered increases. This is a result of higher frequencies being more attenuated than lower frequencies when considering a greater distance traveled by the wave.

## 3. Results and Discussion

In the first part of the study, corresponding to the joint analysis of white and red wines, only the results related to the analysis of wave propagation velocity are presented. Both the frequency analysis and the attenuation study failed to produce conclusive results, which is possibly because the acquired samples varied in several parameters, such as vintage, alcohol content, grape varieties, sugar content, and barrel aging. In the second part of the study, concerning the analysis of single-varietal samples of red wines, the results corresponding to all analyzed parameters related to velocity, attenuation, and frequency components are presented.

### 3.1. Characterization of White and Red Wines

**Figure 2:**
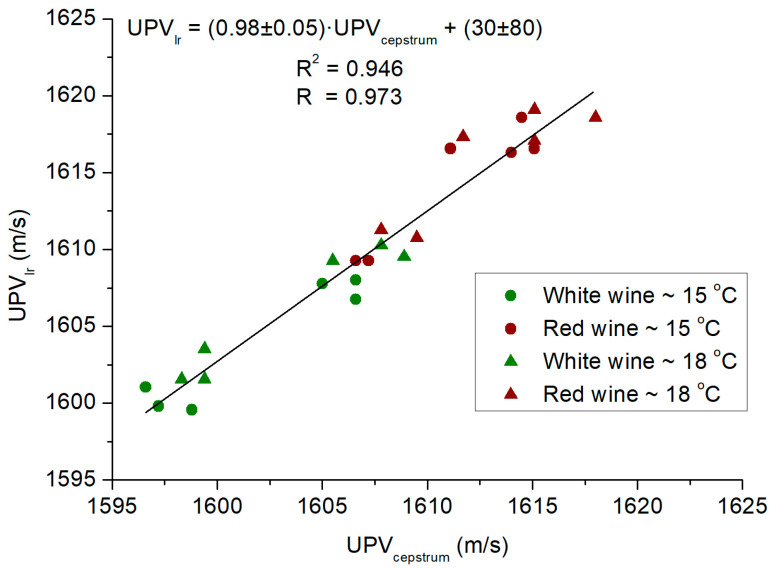
Progression of the average ultrasonic velocity values (*UPV_lr_* and *UPV_cepstrum_*) obtained through both methods across samples inspected at varying temperatures alongside linear regression fitting between them.

Figure 2 depicts the correlation between the mean velocity outcomes derived from both methodologies (*UPV_lr_* and *UPV_cepstrum_*) for the examined specimens across varying temperatures. Ordinary Least Squares (OLS) regression was applied to calculate the correlation coefficient (Pearson’s R) using the XLSTAT software package (version 16.0, Addinsoft, Paris, France). The robustness of the fit was evaluated based on the consistency and strength of the linear relationship, which was indicated by the proximity of the R value to unity. Notably, a robust linear correlation is apparent between the two (R = 0.973; R^2^ = 0.946). Additionally, as anticipated, the slope value approximates unity with an intercept value at the origin that encompasses zero. This finding underscores the credibility of the employed methodologies and facilitates the evaluation of outcomes relying solely on either *UPV_lr_* or *UPV_cepstrum_*.

**Figure 3:**
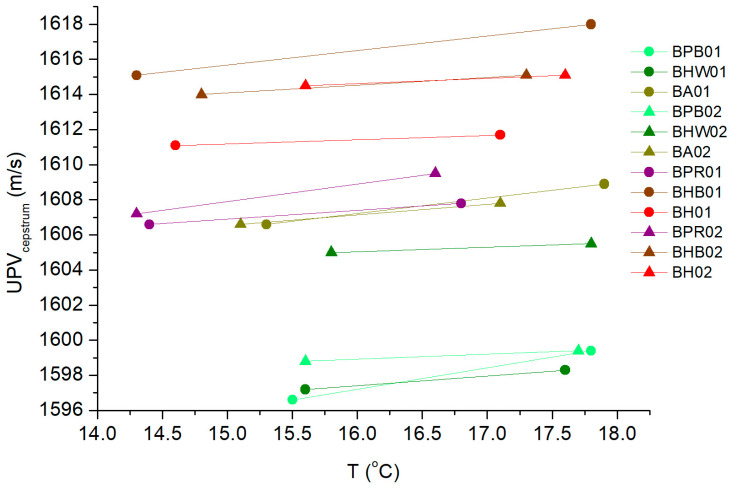
Evolution of mean ultrasonic velocity values (*UPV_cepstrum_*) with temperature for the samples of white and red wine inspected in the initial phase of the study. The green shades have been used for white wines, and the reddish tones have been used for red wines.

Hence, Figure 3 illustrates the temperature variation in *UPV_cepstrum_* values for batches of red and white wines. Initially, it is observed that the velocity outcomes align with those reported in the literature. Thus, using a 1 MHz transducer, Novoa-Díaz et al. establish velocity values ranging from 1550 to 1570 m/s for samples crafted from a hydroalcoholic solution (12% alcohol), a base wine for turbidity testing, and ultimately, a batch of base wines (Merlot variety), in conjunction with DL-Malic Acid and L(+)-Lactic Acid [13]. On the other hand, Lamberti et al., using a 1 MHz transducer too, indicate velocities within the range of 1520–1610 m/s in solutions of saccharose in water and ethanol in water at the concentration levels present during the fermentation process [27]. Van Sint Jan et al. reported velocity values ranging from 1480 to 1620 m/s in solutions prepared with distilled water, sucrose (table sugar) and disinfectant alcohol (96% *v*/*v* ethanol and 4% *v*/*v* methanol), using transducers operating at frequencies of 54 kHz, 500 kHz and 1 MHz [28].

It is noteworthy that all samples exhibit a consistent pattern of increasing ultrasonic propagation velocity as temperature rises. This observation aligns with findings reported in the previous literature [13]. However, such behavior contrasts with that exhibited by other food substrates, such as oils [29] and honey [30,31]. Moreover, a distinct higher velocity value is observed in red wines compared to whites. Thus, despite the samples in this study being crafted from blends of different grape varieties, undergoing various production processes, and possessing different alcohol content, ultrasonic velocity emerges as a valid parameter for classifying and distinguishing red from white wine. Initially, this fact might seem anecdotal, as it is logical to assume that a visual inspection alone would suffice to determine if a wine is red or white. However, this finding already hints at the potential validity of non-destructive ultrasonic inspection in wine sample characterization. In any case, it is worth mentioning that the studies conducted on attenuation and frequency component parameters in these samples do not show such a level of categorization detail.

### 3.2. Characterization of Monovarietal Wines

**Figure 4:**
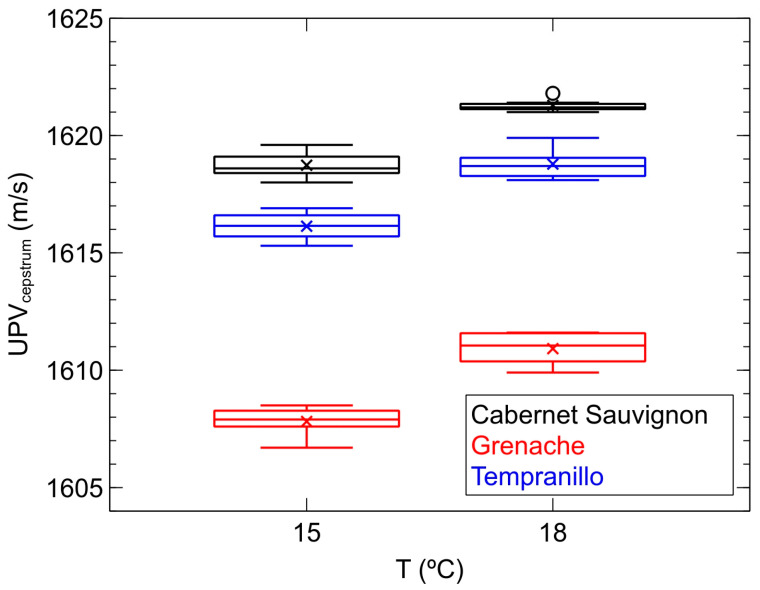
Box-and-whisker plots of the evolution of ultrasonic velocity values (*UPV_cepstrum_*) with temperature for each of the monovarietal samples of red wine inspected in the second phase of the study.

Figure 4 illustrates, in the form of box-and-whisker plots [32], the evolution of propagation velocities (*UPV_cepstrum_*) of ultrasonic waves in the monovarietal wine samples described for the second part of the study, namely those crafted from the Grenache grape variety, those from Tempranillo, and those from Cabernet Sauvignon. For each temperature, the propagation velocities of 50% of the inspected wine samples for each variety lie within the limits of the box, which is intersected by a horizontal line indicating the median velocity obtained. The remaining velocity values either fall within the vertical lines extending from the boxes, representing 25% in the upper part and 25% in the lower part, or are depicted as isolated points, indicating the presence of unusually large or small values relative to the distribution of the other results.

Firstly, the trend previously evidenced in the first part of the study is observed: the increase in sound propagation velocity with the temperature of the samples. Secondly, it is worth noting that the range of velocities obtained fits perfectly within the range established for red wines in the first part of the study with the current range being 1606–1620 m/s at 15 °C and 1610–1622 m/s at 18 °C. Thirdly, and most importantly, the perfect classification established by the velocity parameter in the inspected samples at both 15 °C and 18 °C is evident. Hence, samples crafted from the Grenache variety have the lowest velocity values, ranging from 1606 to 1609 m/s at 15 °C and from 1610 to 1612 m/s at 18 °C, while those crafted from Cabernet Sauvignon exhibit the highest values, specifically ranging from 1618 to 1620 m/s at 15 °C and from 1621 to 1622 m/s at 18 °C. In between are those from Tempranillo, with values ranging from 1615 to 1617 m/s at 15 °C and from 1618 to 1620 m/s at 18 °C. However, the indicated intervals for each variety and temperature do not overlap. It is worth noting that the production process and alcohol content of these wines are identical, differing only in the grape variety chosen for production. Therefore, we can conclude that ultrasonic velocity constitutes a valid parameter for characterizing these samples at either of the two selected temperatures.

**Figure 5:**
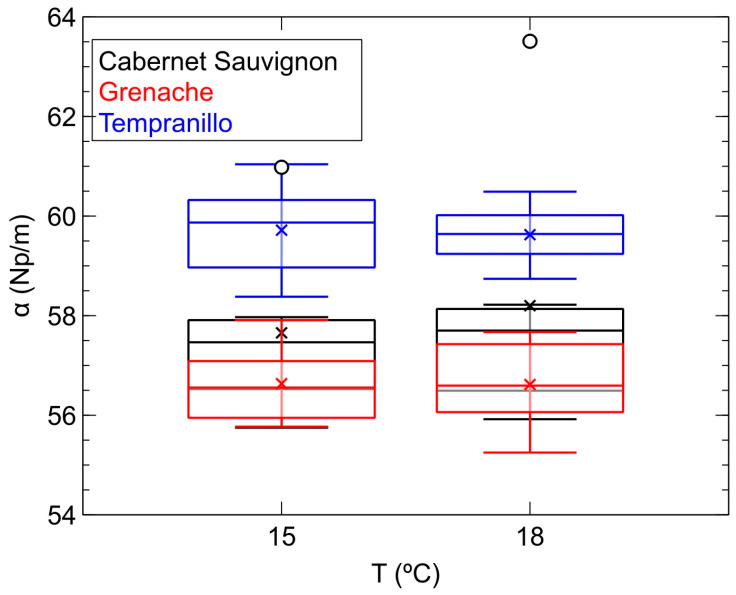
Box-and-whisker plots of the evolution of ultrasonic attenuation (*α*) with temperature for each of the monovarietal samples of red wine inspected in the second phase of the study.

Figure 5 depicts the variation in ultrasonic attenuation (*α*) with temperature. Unlike the behavior observed with velocity, ultrasonic attenuation does not show a clear upward trend with temperature; rather, its value appears to remain stable, at least within the ranges of velocity and temperatures studied.

With regard to the ability of attenuation to classify the studied samples, this parameter only clearly distinguishes those crafted from the Tempranillo variety. These are precisely the most attenuating, particularly when inspected at 18 °C. Below them are those crafted from the other two varieties, Grenache and Cabernet Sauvignon, which have similar attenuation values ranging between 55 and 58 Np/m. As can be inferred from Figure 4 and Figure 5, both ultrasonic parameters, velocity and attenuation, do not exhibit a simple mathematical relationship with each other, thus determining both can provide complementary data of interest when classifying the samples. In our study, it seems evident that velocity emerges as a more sensitive parameter than attenuation when characterizing the samples based on the variety used for their production, as it allows for the separation of the three varieties, whereas attenuation only achieves this with one.

**Figure 6:**
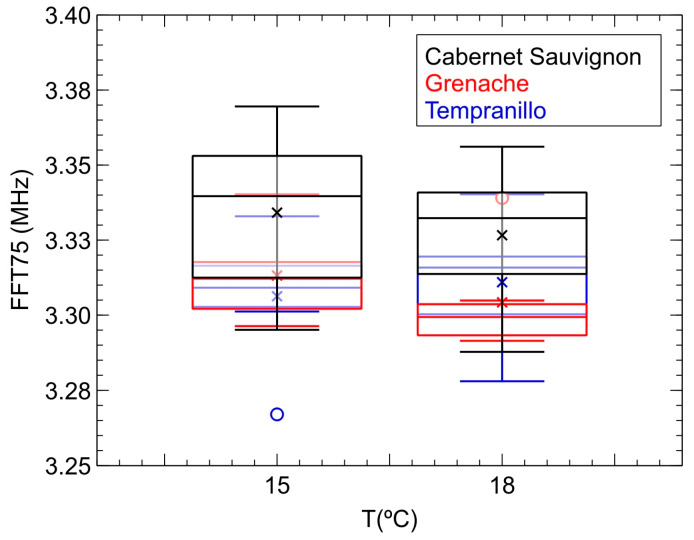
Box-and-whisker plots showing the evolution with temperature of the frequency percentile 75 (*FFT75*) for each of the monovarietal samples of red wine inspected in the second phase of the study.

We finally demonstrate the contribution of frequency components when authenticating the three grape varieties used in the wine samples. Firstly, Figure 6 depicts, in box-and-whisker diagrams, the evolution of the *FFT75* parameter corresponding to the complete A-scan of the received signal. As can be seen, there is also no clear trend in its behavior with temperature, especially considering the ranges obtained for each variety. Additionally, it can also be clearly observed that these ranges completely overlap when considering the three varieties analyzed. The behavior for *FFT25*, *FFT50* and *FFT99* is very similar. At first glance, we could then consider that the analysis of the frequency components propagated in the samples is not a valid parameter for classifying them by grape variety.

**Figure 7:**
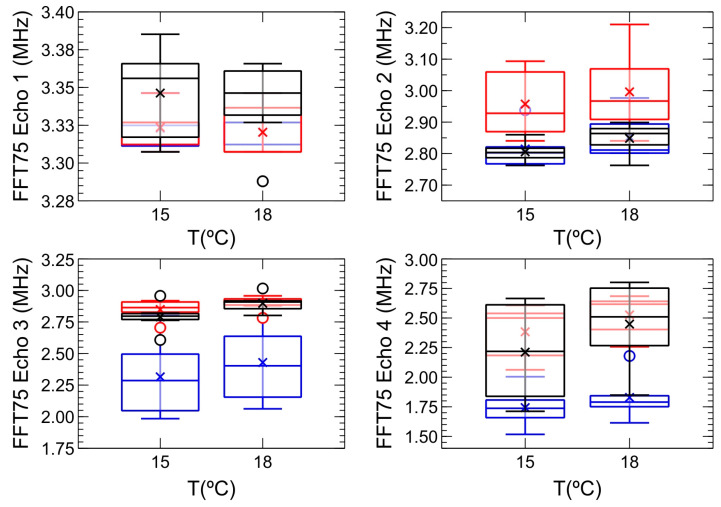
Box-and-whisker plots showing the evolution with temperature of the frequency percentile 75 (*FFT75*) in each of the echoes obtained in the A-scan for each of the monovarietal samples of red wine inspected in the second phase of the study. The color code used is identical to that used in Figure 4, Figure 5 and Figure 6.

However, the analysis of this same parameter, *FFT75*, for each of the four echoes observed in the A-scan of the inspections carried out offers a different perspective. Figure 7 then shows the evolution of *FFT75* with temperature for each of the mentioned four echoes. Thus, although there is still no clear trend regarding the evolution of the frequency components with temperature, there are other aspects that are evident:

- Firstly, there is a clear observation of a decrease in the value of the frequency components as the more distant echoes are considered. This is an expected result, as the signal from the farther echoes has traveled a longer distance and, consequently, its frequency components have been attenuated more. In the same vein, it is evident that the main contribution to the frequency spectrum of the complete A-scan would correspond to the signal from echo 1, with the contribution of the farther echoes, echo 3 and particularly echo 4, being heavily masked. In fact, if we compare the box-and-whisker plots corresponding to the complete A-scan (Figure 6) and echo 1 (Figure 7a), their values are indeed similar. However, when this comparison is made between the complete A-scan and the corresponding echo 3 (Figure 7c) or echo 4 (Figure 7d), the similarity is practically nonexistent.
- It is precisely the study of the *FFT75* behavior in each echo that allows for the classification of the grape variety used in the wine production. Thus, in echo 2, differences between the frequency components of the Grenache variety and the other two varieties begin to emerge, in the sense that it appears that the attenuation of the frequency components in the samples made with this variety is less significant than that of the other two. However, it is in echo 3, particularly noticeable at 15 °C, where the distinct evolution of the attenuation of the frequency components allows for the classification of the three varieties, with the samples made with the Tempranillo variety showing the highest attenuation and those made with Grenache showing the least.

## 4. Conclusions

The research findings, even though obtained from a limited number of samples, suggest the validity of ultrasonic inspection as a reliable non-destructive method for authenticating and characterizing wine samples according, at least, to the grape variety used in their production.

Regarding the ultrasonic wave propagation velocity, the results indicate an increase with the inspection temperature. Overall, the velocity values obtained in wine samples made with the Grenache variety are clearly lower than those made with Tempranillo, and the latter are lower than those made with Cabernet Sauvignon.

As for the attenuation, its behavior with temperature is not as evident: its value remains stable within the inspected range. In this case, only the samples made with the Tempranillo variety stood out from the others, exhibiting a higher attenuation of the propagated ultrasonic waves.

Finally, the behavior of frequency percentiles with temperature requires a more specific study. In this way, frequency analysis of the complete signal does not allow for the categorization of any of the samples made from the different varieties. However, when this frequency analysis is carried out for each of the echoes, the different attenuations experienced by the frequency components depending on the distance traveled, that is, the echo considered, do allow for the almost unequivocal classification of the three varieties studied in the production of the inspected wine samples. In particular, the third echo shows that higher frequencies are more attenuated by samples made from the Tempranillo variety, with those made from Grenache being the least attenuated.

As inferred from the results, it is possible to characterize wine samples based on the grape varieties used in their production with the additional advantage of doing so in a non-destructive manner.

## Figures and Tables

**Table 1:** Origin of the wine samples inspected by ultrasound.

Sample Identifier	Brand	Bottle No.
BPB01	Balancines Punto Blanco	1
BPB02	Balancines Punto Blanco	2
BHW01	Balancines Huno White	1
BHW02	Balancines Huno White	2
BA01	Alunado	1
BA02	Alunado	2
BPR01	Balancines Punto Rojo	1
BPR02	Balancines Punto Rojo	2
BHB01	Balancines Huno Blend	1
BHB02	Balancines Huno Blend	2
BH01	Balancines Haragán	1
BH02	Balancines Haragán	2
T1-1	Finca Antigua Tempranillo	1
T1-2	Finca Antigua Tempranillo	1
T2-1	Finca Antigua Tempranillo	2
T2-2	Finca Antigua Tempranillo	2
T3-1	Finca Antigua Tempranillo	3
T3-2	Finca Antigua Tempranillo	3
C1-1	Finca Antigua Cabernet Sauvignon	1
C1-2	Finca Antigua Cabernet Sauvignon	1
C2-1	Finca Antigua Cabernet Sauvignon	2
C2-2	Finca Antigua Cabernet Sauvignon	2
C3-1	Finca Antigua Cabernet Sauvignon	3
C3-2	Finca Antigua Cabernet Sauvignon	3
G1-1	Finca Antigua Garnacha	1
G1-2	Finca Antigua Garnacha	1
G2-1	Finca Antigua Garnacha	2
G2-2	Finca Antigua Garnacha	2
G3-1	Finca Antigua Garnacha	3
G3-2	Finca Antigua Garnacha	3

**Table 2:** Main transducer characteristics.

Model	Diameter (mm)	Frequency (MHz)	−6 dB Bandwidth (%)	*λ* (mm)	*N* (mm)	*ρ* (°)
Panametrics V381	19	3.50	66.23	0.5	201	1.8

The ultrasonic propagation velocity *UPV* = 1580 m/s was taken for the calculation of *λ*, *N* and *ρ*. *λ*: wavelength. *N*: near-field length. *ρ*: beam angle.

## Data Availability

The datasets used and/or analyzed during the current study are available from the corresponding author on reasonable request.

## Supplementary Materials

The following supporting information can be downloaded at https://www.mdpi.com/article/10.3390/s24134294/s1, Figure S1: A-scan corresponding to the test carried out on a sample of wine with TI = 1000 µs; Figure S2: FFT of the signals received in the inspection shown in SM1; Figure S3: Cepstrum corresponding to the FFT shown in SM2. The t moment corresponding to the periodic excitations of the FFT is shown; Figure S4: Fast Fourier Transforms (FFTs) generated from the A-scan in SM1 for each of the four echoes displayed; Figure S5: Cumulative frequency periodograms correspond to the FFTs shown in SM4. The 25th, 50th, and 75th percentiles of the frequencies are explicitly indicated for each periodogram.
