# Supplementary material for: Use of Non-Destructive Ultrasonic Techniques as Characterization Tools for Different Varieties of Wine
Source: Sensors (Basel). 2024 Jul 2;24(13):4294. doi: 10.3390/s24134294 (PMC11244362; doi:10.3390/s24134294)

Figure S1. A-scan corresponding to the test carried out on a sample of wine with TI=1000 µs.

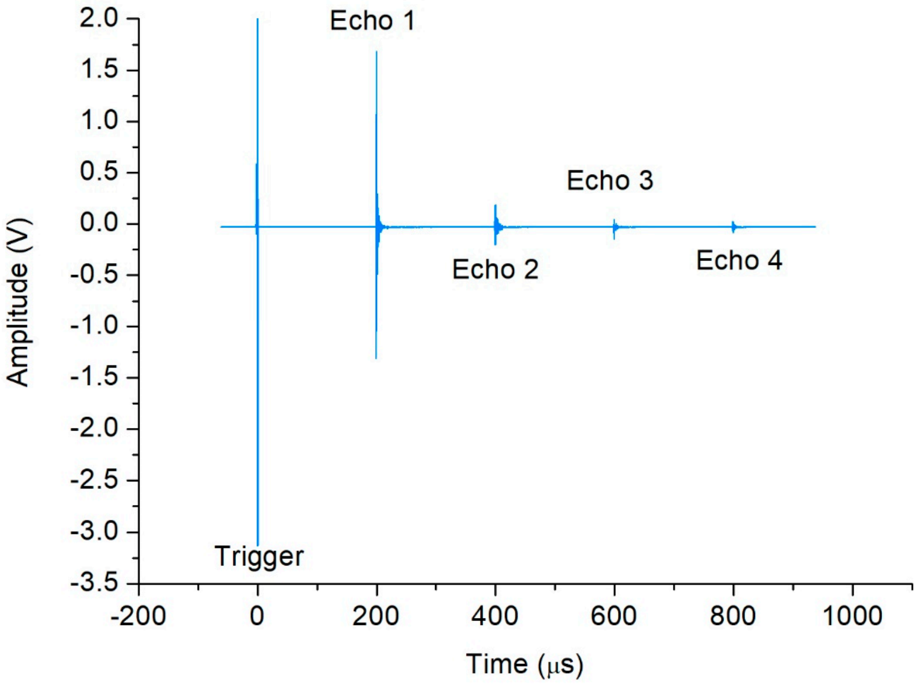

Figure S2: FFT of the signals received in the inspection shown in S1.

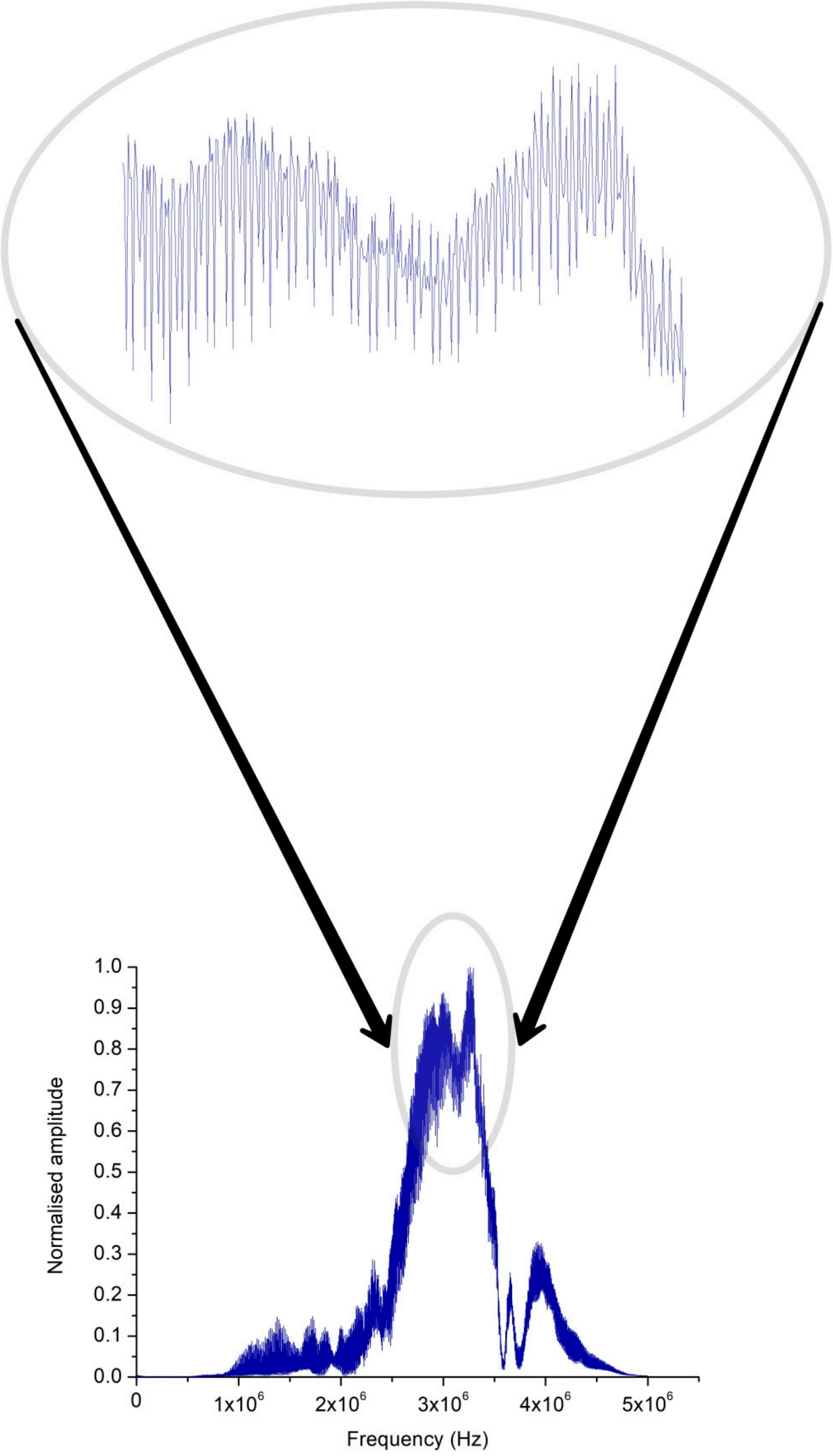

Figure S3: Cepstrum corresponding to the FFT shown in S2. The *t* moment corresponding to the periodic excitations of the FFT is shown.

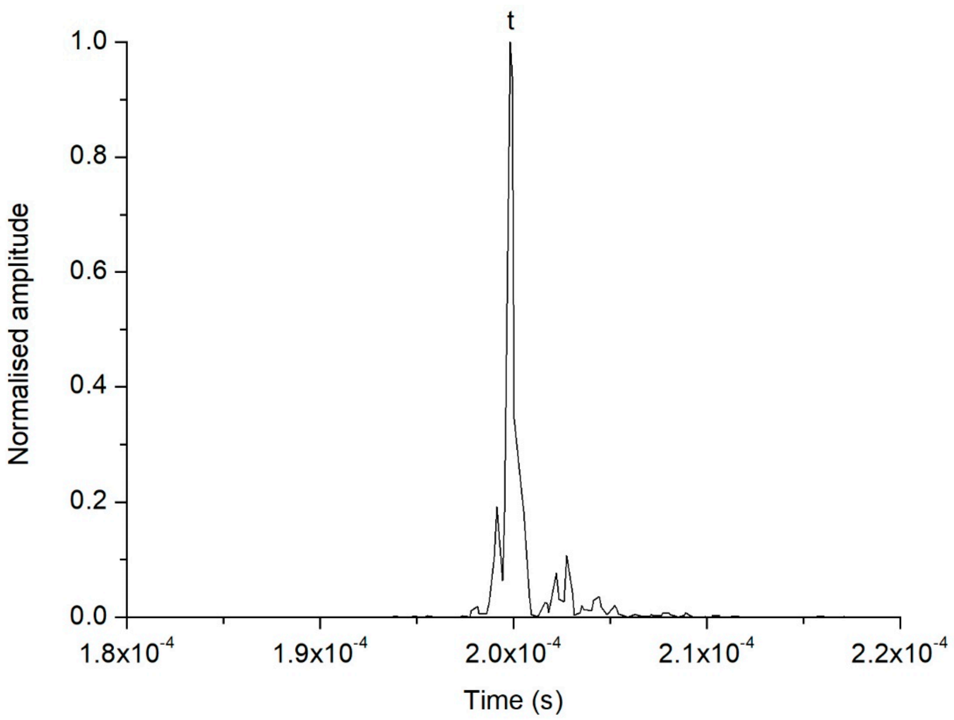

Figure S4: Fast Fourier Transforms (FFTs) generated from the A-scan in S1 for each of the four echoes displayed.

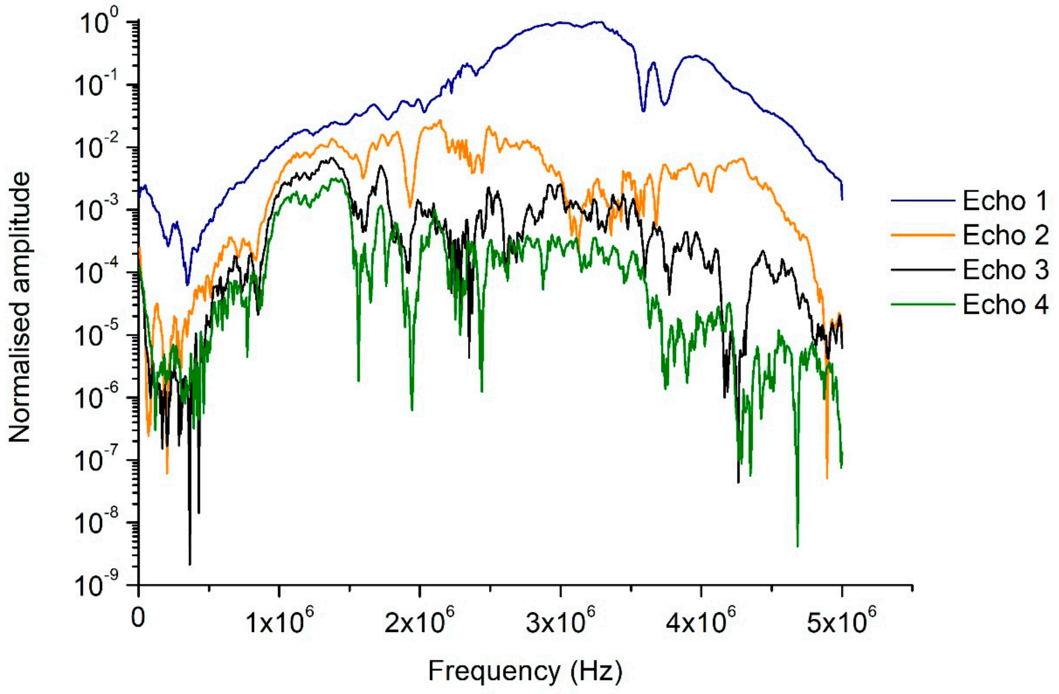

Figure S5: Cumulative frequency periodograms correspond to the FFTs shown in S4. The 25th, 50th, and 75th percentiles of the frequencies are explicitly indicated for each periodogram.

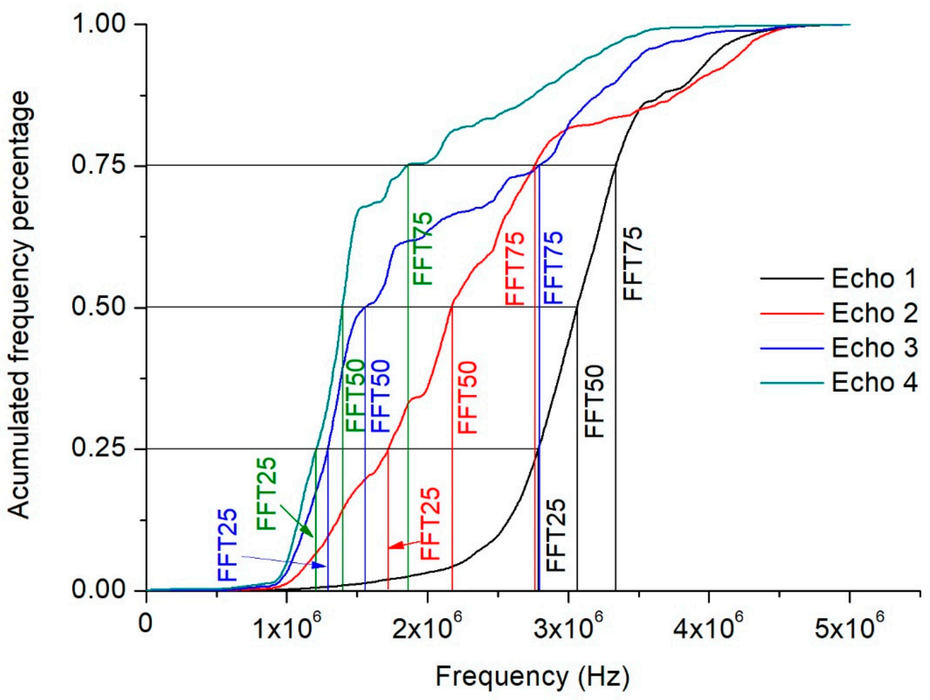

Supplement: Supplementary file 1 [file sensors-24-04294-s001.zip › sensors-3032706-supplementary.pdf]
